# Effect of positive end-expiratory pressure levels on splanchnic perfusion and intra-abdominal pressure: a prospective clinical-experimental study in lung-healthy patients

**DOI:** 10.1186/s44158-026-00411-w

**Published:** 2026-05-19

**Authors:** L. O. Harnisch, D. Tampe, J. Wieditz, O. Moerer

**Affiliations:** 1 Department of Anesthesiology, University Medical Centre Göttingen, Robert-Koch-Str. 40, 37099 Göttingen, Germany; 2 Department of Nephrology and Rheumatology, University Medical Centre Göttingen, Robert-Koch-Str. 40, 37099 Göttingen, Germany; 3 Department of Medical Statistics, University Medical Centre Göttingen, Humboldtallee 32, 37073 Göttingen, Germany

**Keywords:** Positive end-expiratory pressure, Splanchnic circulation, Intra-abdominal pressure, Venous stasis, Venous impedance index, Arterial resistive index

## Abstract

**Background:**

High levels of airway pressure and especially positive end-expiratory pressure (PEEP) have been reported to reduce splanchnic perfusion and consequently lead to organ damage. We evaluated the effect of increasing PEEP levels on splanchnic perfusion and intra-abdominal pressure in a prospective clinical-experimental trial.

**Methods:**

In 20 neurosurgical patients, who were admitted to ICU due to the risk of postoperative complications, we measured the renal and splenic Doppler resistance index (RRI and SRI), intra-abdominal pressure (IAP), and the venous impedance index (VII) at different levels of PEEP (5 to 15 cmH_2_O) using a non-invasive ultrasound technique.

**Results:**

Twenty patients were included in the final analysis. All RRI and SRI values remained within the reference range throughout the protocol, with low measurement variability (SD: RRI 0.06; SRI 0.09; VII 0.22). As PEEP levels increased from 5 to 15 cmH₂O, peak and plateau pressures rose significantly, while driving pressure remained constant. Differences between PEEP levels were not significant for IAP (*η*^2^G = 0.011, *p* = 0.298), RRI (*η*^2^G = 0.034, *p* = 0.253), SRI (*η*^2^G = 0.008, *p* = 0.719), or VII (*η*^2^G = 0.015, *p* = 0.070). No significant correlations with PEEP were found for RRI (*r* =−0.179, *p* = 0.081) or SRI (*r* = −0.130, *p* = 0.205). Mean arterial pressure was stable (84 ± 11 mmHg) and did not correlate with RRI (*r* = 0.195, *p* = 0.106), but correlated with SRI (*r* = 0.250, *p* = 0.037) and IAP (*r* = 0.275, *p* = 0.022). Exploratory mixed-effects models suggested a modest effect of PEEP on RRI (−0.002, *p* = 0.036) and a significant effect on VII (−0.007, *p* = 0.003), though patient-specific factors dominated the variance (*R*^2^c = 0.60–0.91).

**Conclusion:**

In our cohort of mechanically ventilated, hemodynamically stable, non–fluid-responsive, healthy patients, we found no effect of increasing PEEP levels on intra-abdominal pressure or surrogates of splanchnic perfusion, but an increase in renal venous drainage at higher PEEP levels; these findings warrant confirmation in larger samples.

**Trial registration:**

The study was registered in the German clinical trials register (DRKS-ID: DRKS00023895 on 15.02.2021).

**Supplementary Information:**

The online version contains supplementary material available at 10.1186/s44158-026-00411-w.

## Introduction

Mechanical ventilation is crucial for various surgeries and intensive care medicine in general [1]. While lifesaving, it can have side effects, including lung damage known as ventilation-associated lung injury (VALI) [2]. In addition to direct effects on the lungs, negative effects on remote organs such as the liver and kidneys have also been described [3–5].

High intrathoracic pressure, especially elevated positive end-expiratory pressure (PEEP), is commonly proposed to reduce cardiac output by raising intrathoracic pressure, which reduces venous return and right ventricular preload and increases right ventricular afterload by elevating pulmonary vascular resistance [6, 7]. Consecutive reduced tissue oxygenation could lead to short-term functional impairment and long-term organ damage [8–13]. Acute kidney injury in invasively ventilated patients, for example, can result from reduced arterial renal perfusion, but also venous congestion due to decreased drainage, caused by high intrathoracic pressure [14–17].

Thus, many models predict reduced stroke volume and cardiac output with higher PEEP, especially in hypovolemic or other preload-sensitive patients, whereas in cardiogenic pulmonary edema PEEP may be beneficial by lowering left ventricular afterload and improving forward flow [6, 8]. These concepts are typically framed in terms of global hemodynamics; their effects on regional circulation, including the splanchnic organs, remain less well defined.

Doppler indices, such as resistive index (RI), are widely used for the non-invasive evaluation of renal and splanchnic perfusion in critically ill and postoperative patients [7, 18–22]. RI reflects resistance and compliance of the downstream microvascular bed and is therefore a sensitive marker for changes in organ perfusion [23, 24]. In renal physiology, increased RI correlates with acute kidney injury, shock, and systemic hemodynamic disturbances [25]. Similarly, the splenic Doppler RI reflects splanchnic circulation and indicates systemic hemodynamic changes and fluid responsiveness, making these indices valuable for monitoring and guiding organ perfusion management.

Although Doppler‑based measurements are influenced by hemodynamics and reflect changes in vascular impedance rather than direct organ blood flow, they align with preserved organ perfusion in hemodynamically stable patients with normal cardiac function [22]. They must, however, always be interpreted within the broader hemodynamic context, recognizing their limits as surrogate markers. To examine these interactions under controlled conditions, we used Doppler ultrasound to assess how different PEEP levels affect intra-abdominal pressure and splenic and renal perfusion indices in patients without abdominal or pulmonary disease. We hypothesized that progressive increases in PEEP would impair splanchnic and renal perfusion, as well as renal venous outflow, as assessed by RRI, SRI, and VII as surrogates. Furthermore, we aimed to demonstrate that these PEEP-induced alterations in perfusion could be reversed through individualized, targeted fluid administration.

## Methods

The study was designed as a prospective clinical-experimental (physiological) study and carried out in the intensive care unit (ICU) of the Department of Anesthesiology of the Göttingen University Medical Centre from February 2021 to June 2022. No formal a priori sample size calculation was performed. Given the exploratory, mechanistic, within-subject design, the primary aim was to characterize individual splanchnic perfusion responses to graded PEEP rather than to power a definitive outcome trial. The sample size of 20 participants lies at the upper end of what has been reported in similar clinical physiological investigations examining the impact of PEEP on regional or splanchnic perfusion. For moderate within-subject effects comparable to those observed in previous research, a paired design including approximately 20 patients was therefore determined by practical feasibility considerations and is generally sufficient to achieve adequate statistical power in exploratory physiological studies [13, 20, 26]. The primary outcome was the resistance indices of kidney (RRI) and spleen (SRI); secondary outcome was renal venous impedance index and intra-abdominal pressure.

### Ethics

Ethical approval for this study (No. 8/11/20) was provided by the Ethical Committee of Georg-August University Göttingen, Germany, on 12 February 2021; all patients gave informed consent to participate in the study. The study was registered in the German Clinical Trials Register (DRKS-ID: DRKS00023895 on 15.02.2021).

### Patient selection

Postoperative neurosurgical patients who were admitted to the ICU due to high risk for complications were included if they arrived in the ICU deeply sedated (RASS -4 to -5), were aged 18–79, and had an arterial line placed for intraoperative monitoring. Exclusion criteria included intra-abdominal hypertension, chronic pulmonary disease, acute respiratory failure, heart disease, kidney issues, or abdominal surgery history.

### Measurements

All measurements were taken by the first two authors (LOH and DT) together following a standardized protocol (for protocol see SDC); in a single patient, the same vessels were consistently used. For all measurements, a high-frequency curvilinear probe (2–5 MHz) was used. The distensibility of the vena cava was evaluated in the subcostal view 3 cm from the right atrium. As resistance progressively increases from the hilar arteries to the more peripheral parenchymal vessels, sampling of RRI and VII was performed at the level of the interlobar arteries, adjacent to the medullary pyramids. Measurements were conducted immediately after admission to the ICU while patients were sedated with continuous propofol infusion without dose-adjustments throughout the measurements. During the measurement period, the patients were kept supine (0°), and the measurements were performed during ongoing controlled ventilation without a standardized respiratory pause; arterial pressure was continuously monitored but recorded only once at the end of each PEEP level to capture the full effect of adaptation. The measurement sequence began with assessing the distensibility of the inferior vena cava [27, 28]. At the same time, PEEP was set at 5 cmH_2_O, and the remaining ventilator settings were as follows and were maintained throughout the process: Volume-controlled ventilation, tidal volume 6 m kg^−1^ ideal body-weight (IBW), delta-pressure < 12 cmH_2_O, FiO_2_ 1.0, respiratory rate as previously established (for protocol see SDC). PEEP levels were selected to cover meaningful ranges in healthy patients; effects are noticeable at levels of 10 cmH_2_O and above, but levels above 15 cmH_2_O are excessive and ethically questionable in healthy patients [6, 7]; we started our protocol at a lowest PEEP level of 5 cmH_2_O, in line with current guidelines that advise against lower PEEP levels in healthy patients. To maintain lung protection and reduce bias, tidal volume was set to 6 ml/kg in volume-controlled mode. Slightly higher airway pressures than in pressure-controlled modes were accepted for healthy lungs, as they remained within safe limits. Normal lung compliance allowed low driving pressures (mean 6 cmH_2_O) to deliver the target tidal volume. We gradually increased PEEP while carefully monitoring for adverse effects to raise transpulmonary pressure (intraalveolar minus intrapleural pressure) to a clearly positive level.

Bilateral renal resistance index (RRI), renal venous impedance index (VII), splenic resistance index (SRI), and intra-abdominal pressure (IAP) were measured. The sequence was repeated, varying only PEEP, which increased by 2 cmH_2_0 steps up to 15 cmH_2_O as per protocol (see SDC for study protocol); each PEEP level was maintained for 10 min. The ultrasound technique was performed as previously described [29, 30]: After visualizing the kidney and spleen via a postero-lateral acoustic window, the interlobar renal arteries and veins and intraparenchymal splenic arteries were identified with color Doppler ultrasonography. At each step, three Doppler measurements were taken from the same vessel, and the final value was the arithmetic mean of these three measurements. Renal Resistance Index (RRI) is calculated as (peak systolic velocity − end-diastolic velocity) / peak systolic velocity [25]. Renal Venous Impedance Index (VII) is calculated as (maximum venous flow velocity − minimum flow velocity) / maximum venous flow velocity from intrarenal venous Doppler signals acquired simultaneously with arterial waveforms in the same interlobar vessels [31]. Splenic Resistance Index (SRI) is calculated using the identical formula as RRI (peak systolic velocity − end-diastolic velocity) / peak systolic velocity, but measured from intrasplenic arterial branches approximately 1 cm past the splenic hilum using color-guided pulsed-wave Doppler [30]. Intra-abdominal pressure was derived by measuring intravesicular pressure as previously described [32]; although neuromuscular paralysis might have yielded more precise IAP measurements, we deliberately avoided it to capture the effect of high transpulmonary pressure on IAP, including reflexive abdominal muscle tension. With deep sedation but no paralysis, we sought to ensure reliable IAP measurements while preserving natural reflexive abdominal activity, and preventing voluntary muscle activation or coughing.

### Statistical analysis

For statistical analyses, we used R (version 4.3.3, R Foundation, Vienna, Austria) and IBM SPSS statistics (version 29.0, Armonk, NY, USA). For primary analysis, we fitted linear mixed models with RRI/SRI and VII as dependent variable, PEEP, and IAP as fixed effects and random individual effect. Model fit was assessed using marginal and conditional *R*^2^ to determine the proportion of variance explained by fixed effects alone and by the full model, respectively. Differences in RRI, SRI, and VII were analyzed by repeated measures ANOVA, RRI and VII both sides averaged for this analysis. Generalized eta^2^ (*η*_G_^2^) measures the total variance linked to the variable of interest, while controlling for other predictors. Repeated measures correlation was used for pairwise correlations, and Wilcoxon test compared differences between right and left sides. Effect sizes are reported with corresponding two-sided 95% confidence intervals, except for generalized eta^2^ where one-sided 95% CIs are reported. For the computation of repeated measures correlation coefficients, the package rmcorr (Bakdash J, Marusich L (2023). rmcorr: Repeated Measures Correlation. The R package version 0.6.0) was used. Missing data were not specifically handled.

This manuscript adheres to the STROBE checklist.

## Results

We included 21 patients in the study; in one patient, the RRI was above the safety margin of 0.75 right at the first measurement despite 500 ml of crystalloid fluid, and therefore this patient was excluded. We finally measured 20 subjects after elective intracranial supratentorial surgery in the ICU. There were equal female and male patients with a mean age of 52 ± 13 years and a median BMI of 27.6 ± 5.0. Lung compliance was normal (71.04 ± 30.92 ml cmH_2_O^−1^ at PEEP 5 cmH_2_O); liver, kidney, and cholestasis markers were also normal, and fluid status was balanced (IVC distensibility index, 0.28 ± 0.17 at PEEP 5 cmH_2_O), consistent with functional euvolemia in patients with normal lung compliance; see Table 1 for details. Other than neurological issues, no organ impairments were noted. Patients received no medications affecting renal or splanchnic resistance, nor vasoactive drugs during measurements. Protocol compliance was maintained.

**Table 1:** Patient characteristics; *VCI-DI* vena cava inferior distensibility index (measured at PEEP 5 cmH_2_O)

**Parameter**	
Age [years], mean ± SD	52 ± 13
Female:male, *n*	10:10
BMI, mean ± SD	27.6 ± 5.0
VCI-DI mean ± SD	0.28 ± 0.17
Serum Creatinine [mg/dl], mean ± SD	0.76 ± 0.16
BUN [mg/dl], median (IQR)	10 (7–14.3)
AST [U/l], median (IQR)	19 (16–24.5)
ALT [U/l], median (IQR)	16 (8.8–20.5)
GGT [U/l], mean ± SD	35.3 ± 30.8
Alkaline phosphatase [U/l], mean ± SD	56.16 ± 16.75

Two patients needed fluid boluses. In one, RRI and SRI were above 0.75 right from the first measurement and remained there despite 500 ml of fluid, so the measurements were stopped per protocol and the patient was excluded from the analysis because no measurements were obtained. The second patient received 50 ml, which reduced the indices below 0.75, allowing measurements to continue without more boluses.

As PEEP levels increased, peak and plateau pressures increased significantly, while the driving pressure (plateau pressure minus PEEP) remained constant (Table 2).

**Table 2:** Respiratory parameters at the different PEEP levels investigated. *PEEP* positive end-expiratory pressure, *Ppeak* peak airway pressure, *Pplat* plateau airway pressure, driving pressure = Pplat – PEEP

	**PEEP 5**	**PEEP 7**	**PEEP 9**	**PEEP 11**	**PEEP 13**	**PEEP 15**	***p*-value**
Ppeak [cmH_2_O], mean ± SD	19.4 ± 2.1	21.7 ± 3.0	22.9 ± 2.2	25.3 ± 2.6	27.5 ± 2.6	29.6 ± 2.8	< 0.001
Pplat [cmH_2_O], mean ± SD	11.64 ± 2.1	13.2 ± 2.6	14.8 ± 2.6	17.2 ± 2.8	19.0 ± 2.9	21.3 ± 2.8	< 0.001
Driving pressure [cmH_2_O], mean ± SD	6.6 ± 2.1	6.2 ± 2.6	5.8 ± 2.6	6.2 ± 2.8	6.0 ± 2.9	6.3 ± 2.8	0.397
Tidal volume [ml], mean ± SD	422.00 ± 59.69	406.58 ± 68.90	414.95 ± 63.23	416.47 ± 61.74	417.12 ± 61.55	414.05 ± 62.95	0.091
Compliance [ml/cmH_2_O], mean ± SD	71.0 ± 30.9	71.7 ± 32.4	73.6 ± 35.7	71.5 ± 36.6	74.1 ± 37.2	61.5 ± 23.6	0.337

All RRI and SRI values recorded in this study were within the reference range described [22]; variability of repeated measurements was low (standard deviations: RRI 0.06; SRI 0.09; VII 0.22; for the measure of individual patient-dispersion, see SDC Table 1). In the fitted models for RRI and SRI, the effect of PEEP was estimated as −0.002, 95% CI [−0.004; −0.0001] (*p* = 0.036) and −0.002, 95%-CI [−0.004; 0.001] (*p* = 0.152) whereas the effect of IAP was 0.002, 95% CI [−0.002; 0.004] (*p* = 0.473) and 0.002, 95% CI [−0.002; 0.006] (*p* = 0.304). The linear mixed-effects models revealed that only a modest proportion of the variance was explained by fixed effects alone (*R*^2^ₘ = 0.02 for each RRI, SRI and VII, respectively), while the conditional *R*^2^ (*R*^2^_c_ = 0.60/ 0.63/ 0.91 for RRI, SRI and VII, respectively) indicated substantial influence from patient-specific factors.

**Fig. 1:**
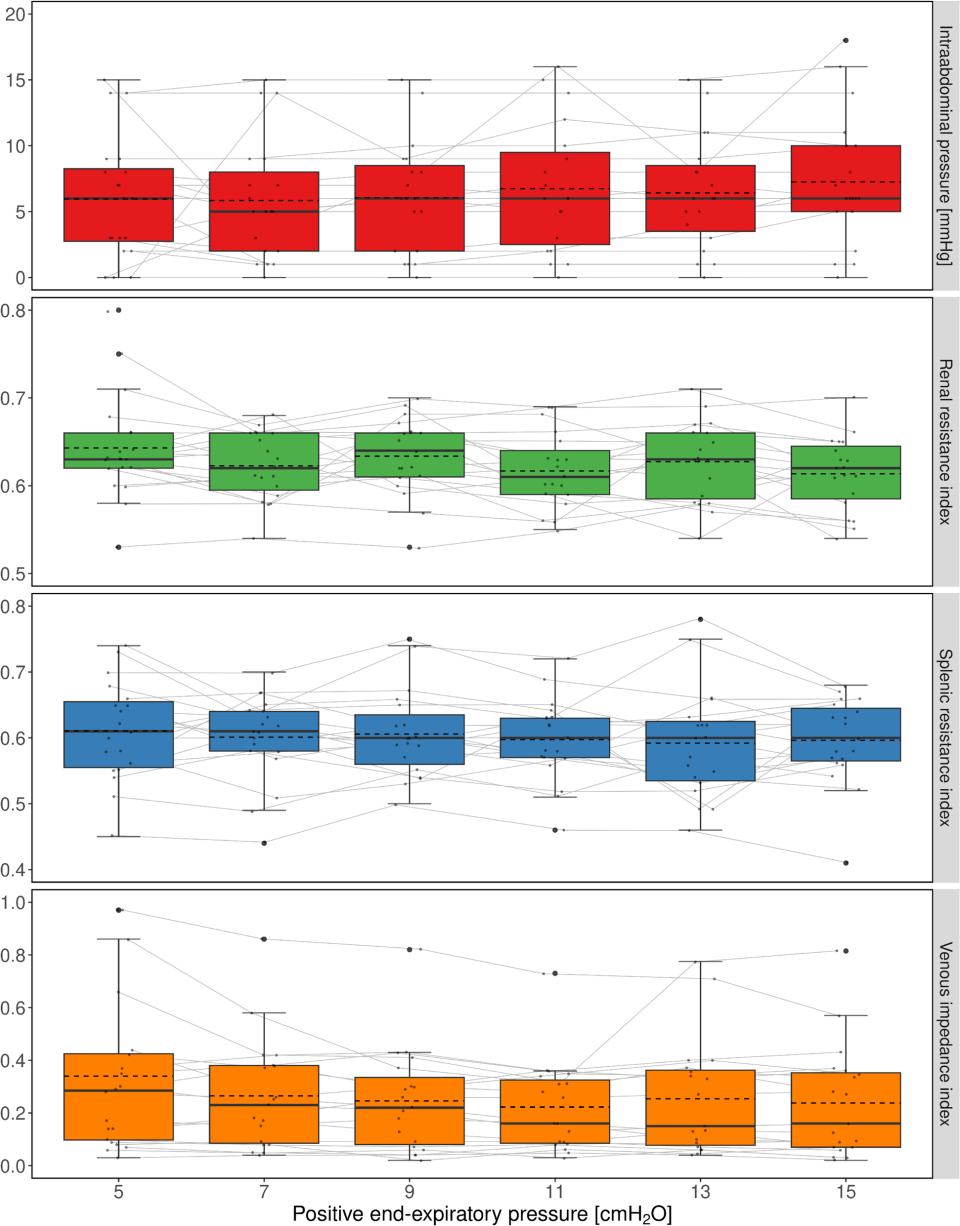
Evolution of intra-abdominal pressure, renal and splenic resistance indices, and venous impedance index over the course of increasing levels of PEEP. Individual data points are expressed as small dots in gray color and outliers as large dots. Data belonging to the same individual were connected by lines

RRI and SRI were correlated, *r* = 0.334, 95% CI [0.144; 0.501] (*p* < 0.001). No significant correlations with PEEP were found for RRI (*r* = −0.179, 95% CI [−0.366; 0.023], *p* = 0.081) or SRI (*r* = −0.130, 95% CI [−0.322; 0.072], *p* = 0.205). Throughout the course of increasing PEEP levels, the differences between PEEP levels were not significant for IAP (*η*_G_^2^ = 0.011, 95% CI [0;1], *p* = 0.298), RRI (*η*_G_^2^ = 0.034, 95% CI [0;1], *p* = 0.253), SRI (*η*_G_^2^ = 0.008, 95% CI [0; 1], *p* = 0.719), or VII (*η*_G_^2^ = 0.015, 95% CI [0;1], *p* = 0.070) (Fig. 1). Moreover, PEEP did not have a significant effect on any of the indices when considered left or right sided.

PEEP significantly influenced VII (−0.007, 95% CI [−0.010; −0.002] (*p* = 0.003), see SDC Fig. 1), unlike IAP, which did not (0.005, 95% CI [−0.002; 0.010] (*p* = 0.175)). The correlation of IAP with VII and PEEP was *r* = 0.053, 95% CI [−0.149; 0.251] (*p* = 0.605) and *r* = 0.213, 95% CI [0.013; 0.397] (*p* = 0.037), respectively.

**Fig. 2:**
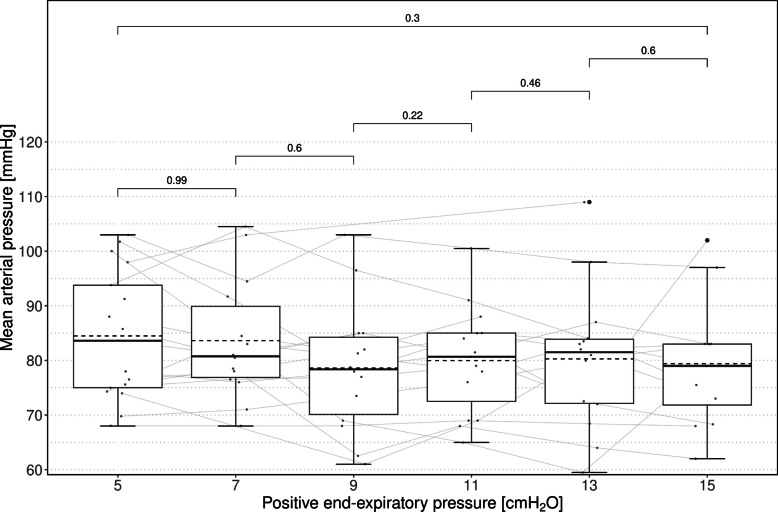
Evolution of mean arterial pressure over the course of increasing levels of PEEP. Individual data points are expressed as small dots connected with gray lines, and outliers are expressed as large dots. Data belonging to the same individual are connected with lines. Means are indicated with dashed lines and compared using paired *t*-tests; unadjusted *p*-values above corresponding brackets

Mean systemic arterial pressures were 84 ± 11 mmHg with minimal variance. No vasopressors were needed. Mean arterial pressure did not correlate with RRI (*r* = 0.195, 95% CI [−0.042; 0.411], *p* = 0.106) but correlated with SRI (*r* = 0.250, 95% CI [0.016; 0.458], *p* = 0.037), VII (*r* = 0.424, 95% CI [0.210; 0.599], *p* < 0.001), and IAP (*r* = 0.274, 95% CI [0.042; 0.478], *p* = 0.022) (Fig. 2 and SDC Fig. 2). There were no significant differences in arterial pressure between neighboring PEEP levels or between PEEP 5 and PEEP 15 (*p* = 0.302).

## Discussion

Our study shows that stepwise PEEP escalation from 5 to 15 cmH₂O does not significantly affect splanchnic vascular impedance or intra-abdominal pressure in hemodynamically stable, functionally euvolemic neurosurgical patients with healthy lungs and abdominal organs. All RRI and SRI values recorded in this study were within the reference range described, with low variability of repeated measurements (SD: RRI 0.06; SRI 0.09; VII 0.22). Throughout the course of increasing PEEP levels, differences between PEEP levels were not significant for IAP, RRI, SRI, or VII. Mean arterial pressure was stable and did not differ between PEEP levels.

Our data do not support earlier reports or theories that higher PEEP (and resulting higher plateau airway pressure) reduces splanchnic perfusion [33–35]. We found no significant effects of PEEP or plateau pressure on splenic or renal resistance indices. With preserved cardiac output and euvolemia, even relatively high PEEP seems well tolerated in patients with normal lungs and abdominal organs. These results, however, do not rule out that the proposed mechanisms (increased right ventricular afterload, reduced venous return, splanchnic hypoperfusion) may be clinically important in critically ill patients with impaired cardiac reserve, severe lung injury, elevated intra-abdominal pressure, or hypovolemia [3, 17]. All patients were hemodynamically stable, with minimal variation in mean arterial pressure, which is consistent with previous findings [8], and MAP did not correlate with RRI (*r* = 0.195, *p* = 0.106), excluding arterial pressure fluctuations as a confounder of renal hemodynamics. Although VII and SRI showed weak-to-moderate correlations with MAP, these occurred within a very narrow, stable pressure range without PEEP-level differences, making their clinical relevance negligible.

We found no statistically significant effect of PEEP on IAP, which aligns with the unchanged resistance indices. Preliminary data from an ongoing trial (DRKS00029328) also indicate that resistance indices, as a surrogate for splanchnic perfusion, remain unchanged despite relevant IAP increases during prone positioning. Thus, splanchnic perfusion appears unaffected by IAP increases that do not meet criteria for intra-abdominal hypertension [36], rendering this statistically significant result clinically irrelevant. Therefore, our results do not support the hypothesis that high intrathoracic pressure increases IAP; instead, even high PEEP levels did not significantly raise IAP or alter splanchnic vascular impedance in hemodynamically stable, non-fluid-responsive patients with healthy lungs and abdomen, even without paralysis.

Autoregulation, often overlooked in prior studies, can obscure the effects of PEEP on organ perfusion and may explain conflicting results. To determine the true effect on splanchnic vascular impedance, a non-autoregulated organ should be examined. The spleen, which has negligible autoregulation [37], is well suited for this purpose, as shown by the correlation between the SRI and mean arterial pressure (MAP), indicating perfusion depends on MAP. Our data also show a strong correlation between renal and splenic resistance indices, supporting the use of either organ. The spleen, however, is preferable because it is singular, requires only one measurement, avoids confounders such as unilateral disease, and lacks autoregulation. We therefore recommend assessing splenic vascular impedance as a surrogate for splanchnic perfusion in clinical and research settings by noninvasively measuring SRI using established cutoffs [22].

Our findings suggest a potential relationship between VII and IAP. A possible explanation for the observed VII decrease at higher PEEP may be the Starling resistor model: when external pressure (IAP) exceeds downstream venous outflow pressure, flow becomes dependent on external pressure, potentially causing vessel collapse and increased resistance [38]. Higher PEEP may raise renal venous outflow pressure enough to prevent collapse against surrounding IAP, thereby reducing venous resistance. Whether this mechanism translates into clinically meaningful improvements—better venous drainage, less congestion, or reduced kidney injury—is unclear and requires further study, especially since RRI remained stable. We acknowledge that VII appears to decrease with PEEP, but we did not systematically assess flow waveforms (continuous vs. discontinuous), so we cannot confirm whether this represents normalization of renal venous hemodynamics or other pressure-related effects. Without direct measurements of venous flow patterns, central venous pressure, and transmural gradients, the exact physiological mechanism remains uncertain and our proposed mechanism is hypothesis-generating.

Although our study focused on splanchnic perfusion, the use stepwise increase in PEEP in our neurosurgical cohort warrants brief consideration of cerebral effects: Current evidence indicates that moderate PEEP (5–15 cmH_2_O) has clinically negligible impact on intracranial pressure (ICP) and cerebral perfusion in patients without severe acute brain injury or intracranial hypertension. A large quantitative analysis of 341 patients and 28,644 paired observations found no significant association between PEEP and ICP at any level in patients without severe lung injury [39]. When ICP does not rise with higher PEEP, multimodal neuromonitoring shows preserved cerebral autoregulation, maintained cerebral perfusion pressure, and stable or improved brain tissue oxygenation [40]. In our elective neurosurgical patients (craniotomy with bone flap replacement, intact dura, no pre-existing intracranial hypertension or mass effect), compliance is preserved, making PEEP transmission and ICP effects even less likely than in patients with impaired intracranial reserve [5]. Thus, the PEEP levels used in our protocol are unlikely to compromise cerebral hemodynamics in this population.

### Limitations

This exploratory, mechanistic study used a feasibility-driven sample size, so all findings are hypothesis‑generating.

We did not test PEEP above 15 cmH_2_O and cannot assess effects beyond this level. Prior literature indicates hemodynamic effects from 10 cmH_2_O becoming excessive at ≥ 15 cmH_2_O; higher PEEP was therefore considered unethical for our cohort.

Because we studied only healthy individuals with normal lung compliance and no abdominal disease, external validity for typical ICU patients—particularly those with ARDS, hypovolemia, shock, obesity, or intra-abdominal hypertension—is limited, which is also a methodological strength, as it removes IAP and low lung compliance as confounders and isolates direct PEEP effects on organ perfusion.

A minor limitation is that one patient received a single 50-mL crystalloid bolus once, to maintain the predefined RRI safety threshold, which may introduce slight interindividual variability. However, its protocolized use reflects best practice and the ethical requirement for safe individualized hemodynamic management. Finally, the short observation period prevents conclusions about long-term effects on splanchnic perfusion.

### Strengths and future directions

The study’s generalizability is supported by the use of noninvasive ultrasound to assess splanchnic perfusion and renal congestion, suitable for broad clinical use, inclusion of patients with healthy lungs and abdomens, providing a baseline for PEEP effects, evaluation of typical PEEP levels (5–15 cmH_2_O) reflecting standard ventilator settings for most elective surgical patients, and the observed correlation between SRI and MAP, validating SRI as a marker of splanchnic perfusion. Future studies should examine longer PEEP exposure, higher-risk populations (ARDS, hypovolemia, intra-abdominal hypertension), and direct venous flow measurements to clarify the mechanisms suggested by the VII findings.

## Conclusion

In our cohort of mechanically ventilated, hemodynamically stable, non–fluid-responsive neurosurgical patients without cardiopulmonary comorbidities, we observed no short-term influence of increasing PEEP levels on intra-abdominal pressure or Doppler-derived indices of splanchnic vascular impedance. In this euvolemic population, PEEP could be adjusted as needed without clear short-term impairment of splanchnic perfusion; however, these exploratory findings cannot be generalized to other patient groups, longer exposures, or different hemodynamic conditions. The possibility that higher PEEP levels reduce venous impedance indices and thereby lessen renal venous congestion needs confirmation.

## Supplementary Information

Supplementary Material 1.

## Data Availability

Data are available from the corresponding author on reasonable request.

## Abbreviations

PEEP: Positive end-expiratory pressure
RRI: Renal Resistance Index
SRI: Splenic Resistance Index
VII: Venous impedance index
VALI: Ventilation-associated lung injury
ICU: Intensive care unit
RASS: Richmond Agitation-Sedation Scale
IAP: Intra-abdominal pressure
MAP: Mean arterial pressure
ARDS: Acute respiratory distress syndrome
