# Supplementary material for: Effect of positive end-expiratory pressure levels on splanchnic perfusion and intra-abdominal pressure: a prospective clinical-experimental study in lung-healthy patients
Source: J Anesth Analg Crit Care. 2026 May 19;6:75. doi: 10.1186/s44158-026-00411-w (PMC13185192; doi:10.1186/s44158-026-00411-w)
Supplement: Supplementary file 1 — Supplementary Material 1. [file 44158_2026_411_MOESM1_ESM.docx]

Study protocol

PEEP 5

10 minutes

IAP, SRI, RRI, VII, ABG

PEEP 7

10 minutes

IAP, SRI, RRI, VII, ABG

PEEP 9

10 minutes

IAP, SRI, RRI, VII, ABG

PEEP 11

10 minutes

IAP, SRI, RRI, VII, ABG

PEEP 13

10 minutes

IAP, SRI, RRI, VII, ABG

PEEP 15

10 minutes

IAP, SRI, RRI, VII, ABG

🡺 RRI > 0.75 🡪 boluses of 50ml crystalloid fluid until RRI again < 0.75, then continue in protocol, maximum bolus volume 500ml

Ventilator settings:

- FiO_2_ 1.0
- Volume-controlled ventilation
- Tidal volume 6ml/kg ideal bodyweight
- Delta-pressure < 12mbar
- Respiratory rate as before

Discontinuation criteria/end of measurement:

- Hemodynamic limitation (RR drop > 20% baseline)
- Hyperinflation of lungs (drop in SpO_2_)
- Intraabdominal pressure > 20mmHg
- Pplat > 30mbar
- 500ml crystalloid applied without influence on RRI
- Last PEEP level reached

**Safety Threshold for RRI**

We selected an RRI > 0.75 as a preemptive safety threshold, rather than lower cutoffs, to intervene as late as safely possible while still preventing progression to clinically significant intrarenal vasoconstriction and minimizing protocol interference. This conservative cutoff is supported by evidence that RRI > 0.75 marks an inflection point beyond which the risk of acute tubular necrosis increases sharply (*Platt JF, Rubin JM, Ellis JH, (1991) Acute renal failure: possible role of duplex Doppler US in distinction between acute prerenal failure and acute tubular necrosis. Radiology 179: 419-423*). That only one patient required a minor fluid bolus further indicates the cohort's excellent baseline volume status.

**Hemodynamic Interpretation of IVC Distensibility Index**

The mean IVC distensibility index was 28% at PEEP 5 cmH₂O, exceeding the classic 18% threshold reported by Barbier et al. in septic patients with acute lung injury (*Barbier C, Loubières Y, Schmit C, Hayon J, Ricôme JL, Jardin F, Vieillard-Baron A, (2004) Respiratory changes in inferior vena cava diameter are helpful in predicting fluid responsiveness in ventilated septic patients. Intensive care medicine 30: 1740-1746*). This value must be interpreted in the context of our integrated hemodynamic data: mean arterial pressure remained stable during PEEP escalation from 5–15 cmH₂O, SRI and RRI as well as IAP were unchanged, and no hemodynamic deterioration or fluid responsiveness occurred with higher PEEP. Together, these findings indicate functional euvolemia - adequate preload and hemodynamic stability in patients with normal respiratory system compliance. Thus, the elevated IVC-DI reflects efficient intrathoracic pressure transmission in compliant lungs rather than preload dependence.

The link between lung compliance and IVC distensibility is well-established. Jardin et al. showed that respiratory system compliance strongly influences intrathoracic pressure transmission: high-compliance lungs (C~L~ >100 mL/cmH₂O) transmitted 37% of airway pressure to the pleural space, whereas low-compliance lungs (C~L~ <30 mL/cmH₂O, ALI/ARDS) transmitted only 24% (p<0.001) (*Jardin F, Genevray B, Brun-Ney D, Bourdarias J-P, (1985) Influence of Lung and Chest Wall Compliances on Transmission of Airway Pressure to the Pleural Space in Critically Ill Patients. Chest 88: 653-658*). This 54% relative increase in pressure transmission in normal-compliance lungs directly accounts for baseline IVC-DI values higher than those reported in critically ill populations with reduced compliance.

**Physiological Rationale for the 10-Minute Observation Period**

Our results show that brief PEEP elevations (10 minutes) do not affect hemodynamics or splanchnic vascular impedance in (functionally) euvolemic neurosurgical patients. The main hemodynamic effects of PEEP arise within seconds to minutes: cardiac output falls to its minimum within 1–2 heartbeats, and respiratory mechanics and gas exchange stabilize within 15–30 minutes (*Berger D, Moller PW, Weber A, Bloch A, Bloechlinger S, Haenggi M, Sondergaard S, Jakob SM, Magder S, Takala J, (2016) Effect of PEEP, blood volume, and inspiratory hold maneuvers on venous return. American journal of physiology Heart and circulatory physiology 311: H794-806*). Thus, our 10-minute observation period is sufficient to capture the acute physiological changes that determine the immediate safety profile of PEEP in this cohort. However, PEEP has time-dependent effects that limit the extrapolation of these findings. Qvist et al. showed that PEEP-induced hemodynamic compromise can persist for at least 8 hours without compensation, and Blevins et al. found that baroreflexes preserve arterial pressure but not cardiac output because passive right ventricular filling remains chronically impaired (*Qvist J, Pontoppidan H, Wilson RS, Lowenstein E, Laver MB. Hemodynamic responses to mechanical ventilation with PEEP: the effect of hypervolemia. Anesthesiology. 1975 Jan;42(1):45–55.*; *Blevins SS, Connolly MJ, Carlson DE. Baroreceptor-mediated compensation for hemodynamic effects of positive end-expiratory pressure. J Appl Physiol. 1999 Jan;86(1):285–93. doi: 10.1152/jappl.1999.86.1.285*). With longer PEEP exposure (hours to days), interindividual variability increases: Kiefer et al. observed no overall change in splanchnic perfusion after 60 minutes of PEEP when cardiac index was stable, yet some patients developed progressively reduced hepatic venous flow, suggesting occult organ hypoperfusion in susceptible individuals (*Kiefer P, Nunes S, Kosonen P, Takala J, (2000) Effect of positive end-expiratory pressure on splanchnic perfusion in acute lung injury. Intensive care medicine 26: 376-383*). Moreover, cumulative PEEP exposure over several days has been linked to a higher incidence of acute kidney injury, likely from progressive worsening renal venous congestion (*Benites MH, Suarez-Sipmann F, Kattan E, Cruces P, Retamal J, (2025) Ventilation-induced acute kidney injury in acute respiratory failure: Do PEEP levels matter? Critical care 29: 130*).

SDC table 1: Patient individual dispersion measures. Each index was determined by calculating the arithemtic mean from three individual measurements with the dispersion measures given in this tabel. RRI = renal resistance index, SRI = splenic resistance index, VII = venous impedance index.

| Patient | Index | Variance | Standard deviation |
| --- | --- | --- | --- |
| 1 | RRI | 0.02 | 0.04 |
|  | SRI | 0.08 | 0.09 |
|  | VII | 0.076 | 0.27 |
| 2 | RRI | 0.01 | 0.02 |
|  | SRI | 0.02 | 0.04 |
|  | VII | 0.005 | 0.07 |
| 3 | RRI | 0.02 | 0.04 |
|  | SRI | 0.01 | 0.03 |
|  | VII | 0.003 | 0.05 |
| 4 | RRI | 0.01 | 0.04 |
|  | SRI | 0.02 | 0.05 |
|  | VII | 0.001 | 0.03 |
| 5 | RRI | 0.02 | 0.04 |
|  | SRI | 0.02 | 0.05 |
|  | VII | 0.007 | 0.08 |
| 6 | RRI | 0.01 | 0.04 |
|  | SRI | 0.10 | 0.10 |
|  | VII | 0.002 | 0.04 |
| 7 | RRI | 0.02 | 0.04 |
|  | SRI | 0.02 | 0.05 |
|  | VII | 0.011 | 0.11 |
| 8 | RRI | 0.02 | 0.05 |
|  | SRI | 0.02 | 0.05 |
|  | VII | 0.024 | 0.16 |
| 9 | RRI | 0.05 | 0.08 |
|  | SRI | 0.03 | 0.05 |
|  | VII | 0.02 | 0.14 |
| 10 | RRI | 0.03 | 0.05 |
|  | SRI | 0.01 | 0.03 |
|  | VII | 0.06 | 0.08 |
| 11 | RRI | 0.02 | 0.04 |
|  | SRI | 0.01 | 0.03 |
|  | VII | 0.01 | 0.10 |
| 12 | RRI | 0.03 | 0.05 |
|  | SRI | 0.04 | 0.07 |
|  | VII | 0.01 | 0.03 |
| 13 | RRI | 0.02 | 0.04 |
|  | SRI | 0.01 | 0.04 |
|  | VII | 0.01 | 0.04 |
| 15 | RRI | 0.05 | 0.07 |
|  | SRI | 0.06 | 0.07 |
|  | VII | 0.02 | 0.04 |
| 16 | RRI | 0.04 | 0.06 |
|  | SRI | 0.02 | 0.04 |
|  | VII | 0.07 | 0.08 |
| 17 | RRI | 0.07 | 0.08 |
|  | SRI | 0.02 | 0.04 |
|  | VII | 0.01 | 0.04 |
| 18 | RRI | 0.02 | 0.04 |
|  | SRI | 0.02 | 0.05 |
|  | VII | 0.04 | 0.06 |
| 19 | RRI | 0.02 | 0.04 |
|  | SRI | 0.02 | 0.04 |
|  | VII | 0.051 | 0.23 |
| 20 | RRI | 0.01 | 0.04 |
|  | SRI | 0.03 | 0.05 |
|  | VII | 0.01 | 0.10 |
| 21 | RRI | 0.04 | 0.07 |
|  | SRI | 0.04 | 0.07 |
|  | VII | 0.044 | 0.21 |


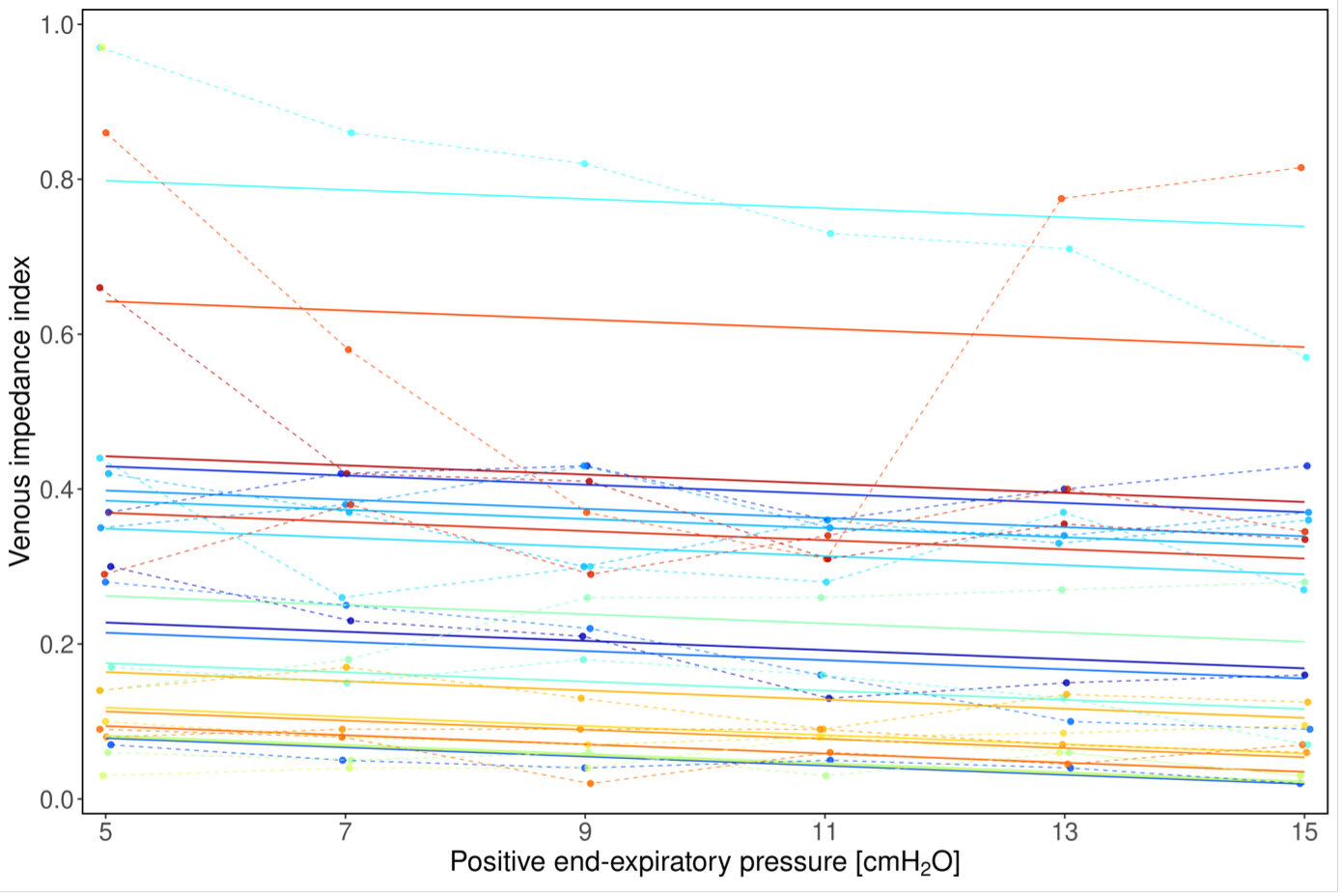


SDC figure 1: Effect of positive end-expiratory pressure on venous impedance index in our cohort in a mixed linear model. Estimated effect for increasing PEEP as solid lines. Individual points are represented as dots of different colors. Data points belonging to the same individual are connected by dotted lines.


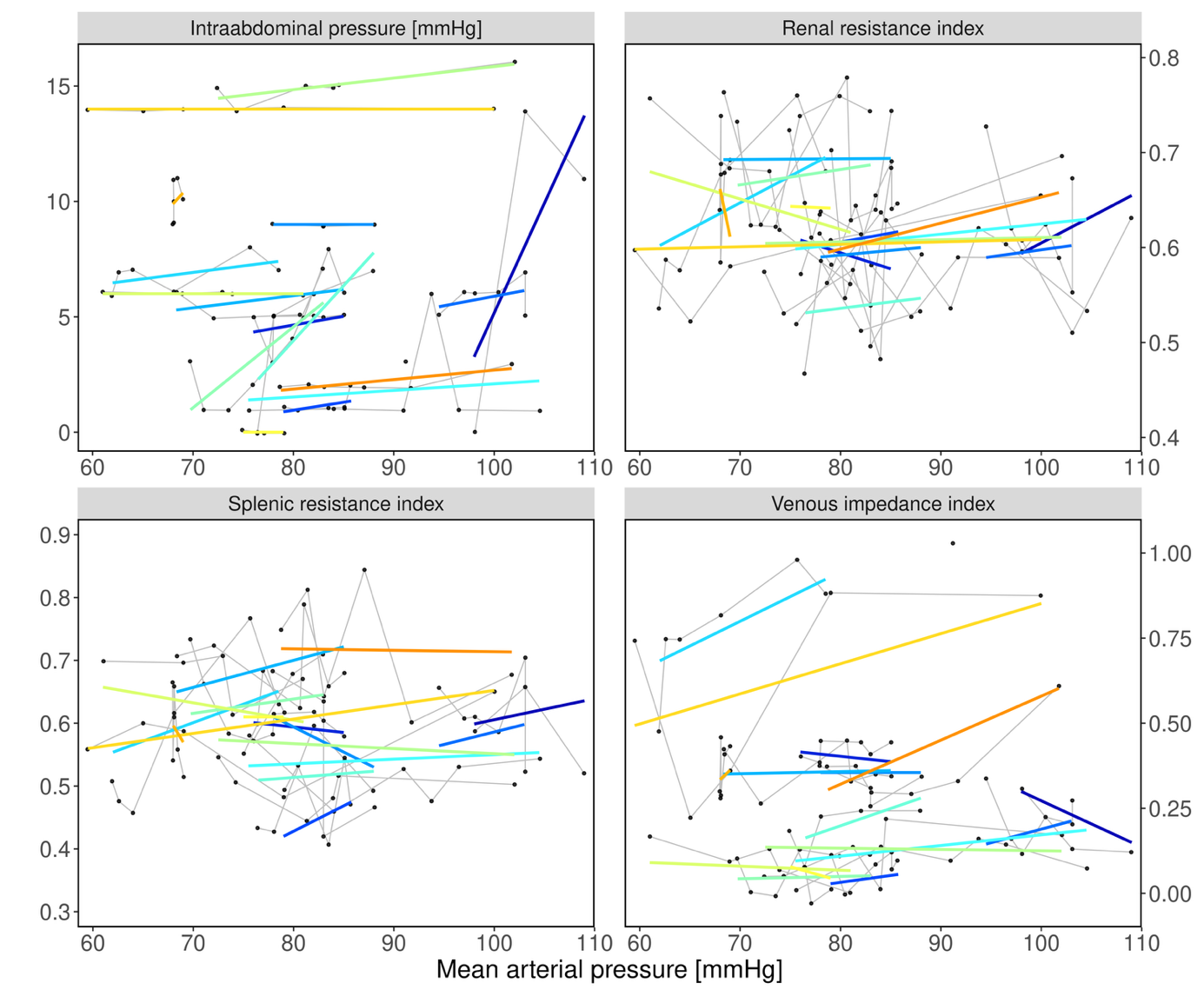


***SDC figure 2:*** Scatter plot of mean arterial pressure vs. Intraabdominal pressure (top left), RRI (top right), SRI (bottom left), and VII (bottom right). Data belonging to the same individual (dots) are connected with black lines. Within-individual regression fit as solid, colored lines. Both variables exhibit significant repeated measures correlation with MAP.
